# Ultra-Short Pulse Generation in a Three Section Tapered Passively Mode-Locked Quantum-Dot Semiconductor Laser

**DOI:** 10.1038/s41598-018-38183-1

**Published:** 2019-02-11

**Authors:** Stefan Meinecke, Lukas Drzewietzki, Christoph Weber, Benjamin Lingnau, Stefan Breuer, Kathy Lüdge

**Affiliations:** 1 Institut für Theoretische Physik, Technische Universität Berlin, 10623 Berlin, Germany; 2 Institut für Angewandte Physik, Technische Universität Darmstadt, 64289 Darmstadt, Germany

## Abstract

We experimentally and theoretically investigate the pulsed emission dynamics of a three section tapered semiconductor quantum dot laser. The laser output is characterized in terms of peak power, pulse width, timing jitter and amplitude stability and a range of outstanding pulse performance is found. A cascade of dynamic operating regimes is identified and comprehensively investigated. We propose a microscopically motivated traveling-wave model, which optimizes the computation time and naturally allows insights into the internal carrier dynamics. The model excellently reproduces the measured results and is further used to study the pulse-generation mechanism as well as the influence of the geometric design on the pulsed emission. We identify a pulse shortening mechanism responsible for the device performance, that is unique to the device geometry and configuration. The results may serve as future guidelines for the design of monolithic high-power passively mode-locked quantum dot semiconductor lasers.

## Introduction

Passively mode-locked semiconductor lasers are photonic light sources, that produce sequences of short equidistant optical pulses at high repetition rates without the need for an external driving frequency^1,2^. They find a multitude of applications in optical data communication^3,4^, metrology^5,6^, medical imaging^7^ and optical clocking^8^. Monolithically integrated semiconductor based designs have the advantages of straight-forward growth and processing while keeping a small footprint, which makes them favorable for future photonic integration^9^. However, spontaneous emission noise and the absence of an external reference clock in such devices leads to relatively pronounced timing and amplitude jitter^10–13^, which are limiting factors for applications. Techniques such as hybrid mode-locking^14–16^, optical injection^17,18^, optical and opto-electronic self-feedback^19–29^ allow to improve the timing stability considerably, but come at the cost of additional electronics and optics, which need to be properly calibrated and controlled. To avoid this, it is therefore highly desirable to optimize the laser design, such that an excellent pulse train stability can be achieved without additional control schemes.

One optimization approach focuses on the device geometry and cavity design, where the precise tuning of the saturable absorber (SA) length^30,31^ and the facet reflectivity of the adjacent facet^32,33^ can lead to shorter pulses and an increased pulse train stability. Moreover, a tapered gain section can lead to an additional pulse shortening and a strong increase in output power^34–37^. Employing semiconductor quantum dots as an active medium comes with advantages such as high differential gain, ultra-fast recovery, broad gain spectra, small chirp and low temperature sensitivity, due to their atom-like discrete energy levels^37–41^. These properties can be employed to generate stable mode-locked pulse trains with sub-ps pulses at high repetition rates^3,34,36,42^. By positioning the absorber section at different cavity positions, the pulse peak power and the mode-locking performance can be improved^43,44^.

In this work, we experimentally and theoretically investigate the optical pulse performance and emission dynamics of a three section tapered semiconductor quantum dot laser with a saturable absorber section positioned at approximately one third of the cavity length. The laser output is characterized in terms of peak power, pulse width and timing and amplitude stability. A semi-classical traveling-wave model excellently reproduces the measurements and is further used to study the spatio-temporal pulse evolution and pulse-generation mechanism.

The paper is organized as follows: Section 2 introduces the device and describes the experimental characterization setup. The results are presented in Sec. 3, which is divided into subsections: The measured and simulated dynamics and performance figures are presented in Sec. 3.1. The pulse-generation and shaping mechanism in the fundamental mode-locking regime is analyzed in Sec. 3.2. The influence of the taper angle is investigated in Sec. 3.3 and the influence of the saturable absorber position is studied in Sec. 3.4. Finally, conclusions are drawn in Sec. 4. Additionally, Sec. 5.1 develops the numerical model and describes the simulation techniques.

## Device and Setup

**Figure 1:**
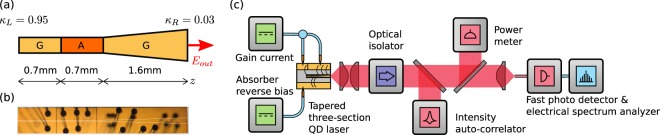
(**a**) Sketch of the three section quantum dot semiconductor laser. The total length of 3 mm corresponds to 13.24 GHz fundamental repetition rate. G denotes the two gain sections and A the reverse biased absorber section. The left facet is high-reflection and the right output facet is anti-reflection coated resulting in *κ*_*L*_ = 0.95 and *κ*_*R*_ = 0.03. *z* denotes the longitudinal dimension of cavity. (**b**) corresponding light microscope picture of the device. (**c**) Experimental setup. A power meter, a nonlinear intensity auto-correlator and an electrical spectrum analyzer are used for the characterization of the dynamics.

The three-section laser consists of 10 layers of InAs quantum dots grown on a GaAs substrate using molecular beam epitaxy. The cavity length amounts to 3 mm corresponding to a repetition rate of 13.24 GHz. The laser has been processed into a 0.7 mm long straight section, a 0.7 mm long absorber section and a 1.6 mm long tapered section with a full taper angle that amounts to 2°. We denote the left side of the saturable absorber as the SA starting position ${z}_{{\rm{SA}}}^{{\rm{s}}}=0.7$ mm. A sketch of the device geometry is shown in Fig. 1(a). The straight section width is 14 *μ*m. Confinement of the optical field in lateral direction is achieved by gain-guiding. In our simulations, we assume an effective active region width of *w*_0_ = 4 *μ*m to approximate the effects of the gain-guided structure, while lateral dimensions are not taken into account. The tapered output facet on the right is anti-reflection coated (AR) resulting in a reflectivity *κ*_*R*_ = 0.03, while the facet on the left is high-reflection coated (HR), resulting in a reflectivity *κ*_*L*_ = 0.95. Figure 1(b) shows a light microscope picture of the laser.

Figure 1(c) shows a schematic, its biasing and the developed pulsed emission characterization setup. Lasing emission is collimated and sent through an optical isolator to prevent unwanted back reflections, which would alter the dynamics of the laser. The analysis of the optical pulse width Δ*t* is performed by a nonlinear intensity auto-correlator and the average optical power *P*_avg_ is obtained by a power meter. After fiber coupling the laser emission, the radio-frequency analysis (pulse repetition frequency *f*_rep_ and repetition linewidth Δ*ν*) is performed by a direct detection configuration using a fast photo-detector connected to an electrical spectrum analyzer. Pulse peak power is estimated by *P*_pk_ = *P*_avg_ * *f*_psf_/(*f*_rep_ * Δ*t*) taking into account an according pulse-shape-factor *f*_psf_^36,45^. In the experiment, the amplitude jitter is quantified by the relative standard deviation of pulse peak power fluctuations and is calculated from the radio-frequency spectrum (electrical bandwidth: 50 MHz to half of the repetition rate)^37,46^. The temporal pulse train stability is quantified by the standard deviation of the pulse-to-pulse timing fluctuations and is estimated from the repetition linewidth Δ*ν*^21,47^. Mode-locking stability is defined as an amplitude jitter below 3% and a pulse-to-pulse timing jitter below 250 fs, which corresponds to 0.33% of the pulse repetition period.

## Results

### Dynamics and Performance

**Figure 2:**
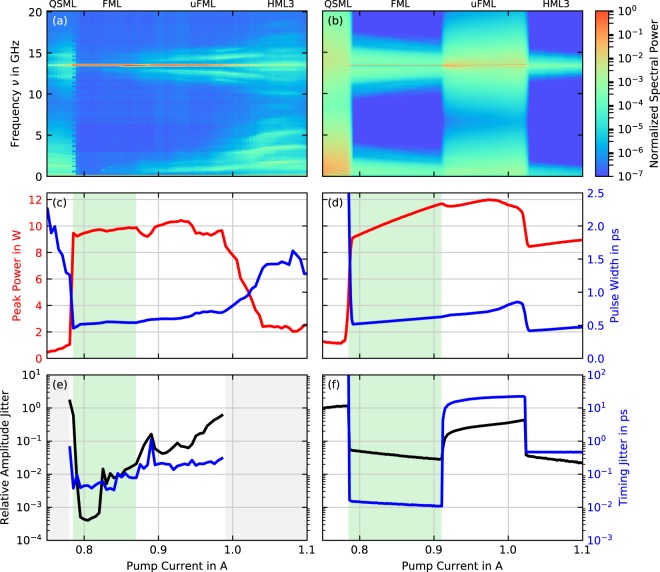
Performance of the QD tapered laser. Measured results are shown on the left and simulated results on the right. (**a**,**b**) Power spectra, with the respective mode-locking state denoted on top, for an increasing pump current: We observe Q-switched mode-locking (QSML), fundamental mode-locking (FML), unstable fundamental mode-locking (uFML) and third order harmonic mode-locking (HML3). (**c**,**d**) Pulse peak power (red) and pulse width (blue) and (**e**,**f**) amplitude (black) and timing jitter (blue). A green background in (**c**–**f**) denotes stable FML.

In this section, we characterize the measured device output in terms of the mode-locking state and pulse performance and compare the results to simulations (see methods section 5.1 for details on the model). We study the laser emission at a reverse bias of *U* = −6V, which yields the best performance figures and scan the pump current. The results are presented in Fig. 2, where the left column shows the measurements and the right column the simulations. The top row shows color coded radio-frequency (RF) spectra, where each spectrum is normalized to its maximum, the middle row shows the pulse peak power (red) and pulse width (blue) and the bottom row amplitude (black) and timing (blue) jitter. Our simulation results are obtained by averaging over 400000 round-trips (≈30 *μ*s) for each pump current.

Scanning the pump current from 750 mA to 1100 mA, we use the RF spectrum Fig. 2(a) and the auto-correlation (AC) signal (not shown) to determine the mode-locking state. As indicated on top of Fig. 2(a), Q-switched mode-locking (QSML) is observed from 750 mA to 780 mA, fundamental mode-locking (FML) from 780 mA to 890 mA, unstable fundamental mode-locking (uFML) from 890 mA to 990 mA and third order harmonic mode-locking (HML3) from 990 mA to 1100 mA. FML and uFML produce a pronounced RF-peak at *ν* ≈ 13.24 GHz, corresponding to the cavity round trip time, while HML3 produces its first RF-peak at *ν* ≈ 40 GHz, which is outside the detection range. QSML and dynamical instabilities lead to increased low-frequency contributions, which can be seen outside the FML region. Peculiarly, the QSML pulses are spaced by a third of round trip, leading to only a small peak at the fundamental frequency at ≈13.24 GHz. We explain the occurrence of this inter-pulse spacing by a colliding pulse mechanism^48^. Two of three evenly spaced pulses meet in the absorber section, which roughly divides the device at the one third position, and thus saturate it more efficiently. Our simulated spectra are plotted in Fig. 2(b) and exhibit the same sequence of mode-locking states for an increasing pump current *P*, thus matching the measurements quite well. Within the HML3 region the simulations indicate an RF spectrum with less instabilities indicating more stable operation. Morever, in the QSML and uFML regions the low frequencies appear at slightly smaller values.

**Figure 3:**
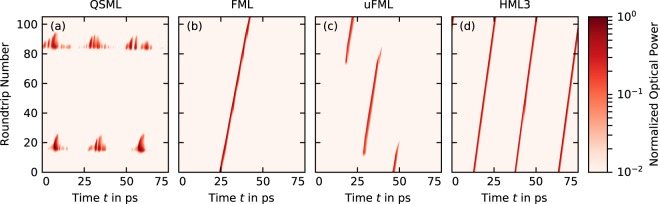
Simulated pseudo space-time plots showing (**a**) Q-switched mode-locking (QSML, *P* = 0.75A), (**b**) fundamental mode-locking (FML, *P* = 0.81A), (**c**) unstable fundamental mode-locking (uFML, *P* = 0.95A) and (**d**) third order harmonic mode-locking (HML3, *P* = 1.08A). Time is cut into slices with the length of the cold cavity round-trip time *T*_0_ = 75 ps and the optical power is color coded and normalized to its respective maximum.

We illustrate the different dynamics for an increasing pump current in Fig. 3 with simulated pseudo space-time plots, where time-series are sliced into pieces with the length of the cold cavity round-trip time and stacked on top of each other to create a color-coded 2D-map of the pulse evolution. Q-switched mode-locking Fig. 3(a) is composed of sets of broad pulses with inter-pulse spacings of about 25 ps, thus the QS-ML is running at the third harmonic frequency, which is also observed in the experiment. The slow envelope has a long period of 5 to 10 *μ*s and leads to the low frequency of about 100 to 200 MHz that we find in Fig. 2(b). Pulse emission ceases in between Q-switched bursts. Fundamental mode-locking is represented by a narrow line in the space-time plot Fig. 3(b) that tilts to the right as the pulse period is slightly longer than the cold-cavity round-trip time. Further increasing the pump current leads to a loss of stability of the FML pulse train and noise induced perturbations create a competing pulse train that periodically ends up taking the gain from the previous pulse train (see Fig. 3(c)). This switch between pulse-trains occurs at round-trip number ≈20 and ≈80 and it takes about 10 round-trips. The period of this pulse-train switching results in the slow frequency of about 150 MHz in the spectrum plotted in Fig. 2(b). Finally, third order harmonic mode-locking Fig. 3(d) resembles FML, except that three pulse-trains are observed within one round-trip.

Turning our focus back to the characterization of the pump current scan, we use a nonlinear intensity auto-correlator and a power-meter data to estimate the pulse width (blue) and pulse peak power (red) as plotted in Fig. 2(c). We find pulses as short as 500 fs with about 10 W peak power in the FML and uFML region. Within that region, the pulse-width increases with the pump current from 500 fs to 600 fs, which we attribute to a faster recovery of the gain due to ultra fast refilling of the GS occupation from the ES^49^. In the QSML region, peak power values are below 1 W and the pulse widths exceed 1.5 ps. In the HML3 region, peak power values drop to ≈2.5 W and the pulse width increases to ≈1.5 ps. Plotted in Fig. 2(d), simulation results excellently reproduce the measurements in the QSML, FML and uFML region. Simulated pulses in the HML3 region carry the same energy but differ from the measurement as they are much shorter (≈500 fs) and consequently higher in peak power (≈8 W). We explain this discrepancy in pulse peak power and width by the difference in pulse train stability, which is evident in the measured and simulated spectra plotted in Fig. 2(a,b). While not being affected by this dynamical instability, simulated pulses in the HML3 region benefit from a colliding pulse mechanism which has been shown to reduce pulse width^48^.

To complement the performance figures, we analyze the pulse-train stability in terms of timing (blue) and amplitude jitter (black) in Fig. 2(e). Due to the bandwidth range of the spectrum analyzer, both quantities can only be evaluated in FML and uFML regime from 780 mA to 990 mA. Between 780 mA and 870 mA, we observe pulse-to-pulse timing jitter values below 100 fs and amplitude jitter values below 2%, thus a 90 mA range of excellent stability, which overlaps with the region of shortest pulses. The degradation of the pulse stability beyond 870 mA comes with the increase of the low-frequency noise and side modes in the spectrum, which we associate with the uFML region. Our simulated amplitude and timing jitter are shown in Fig. 2(f), where the same qualitative behavior is reproduced. Quantitatively however, the results differ as the timing and amplitude jitter are directly computed from the pulse distribution within the time series and not as in the experiment indirectly from the RF-spectrum. Hence, we obtain a timing jitter between 10 fs and 16 fs and amplitude jitter between 3% and 6% in the FML region, followed by a large jump to timing jitter above 1 ps and amplitude jitter above 20% in the uFML region. Moreover, in the QSML region, we obtain very large timing and amplitude jitter values as expected. In the HML3 region we find the amplitude jitter to be comparable to the FML region, while the timing jitter is at ≈450 fs almost two orders of magnitude larger than it is in FML region.

### Ultra-Short Pulse Generation

In order to study the underlying mechanisms involved in the generation of short and stable pulses in the FML regime (810 mA), we use simulations to investigate the internal carrier dynamics of the laser and focus on the pulse evolution along one round-trip. Thus, we adapt the co-moving frame, in which the new time *t*′(*z*) is constant along the propagation of a small perturbation within the cavity.

1 $$\begin{array}{ccc}{t}^{{\rm{^{\prime} }}}(z) & = & \{\begin{array}{cc}{t}^{-}(z)=t+\frac{z}{{v}_{g}} & {\rm{f}}{\rm{o}}{\rm{r}}\,{E}^{-}(z,t)\\ {t}^{+}(z)=t-\frac{z}{{v}_{g}}-\frac{l}{{v}_{g}} & {\rm{f}}{\rm{o}}{\rm{r}}\,{E}^{+}(z,t)\end{array}\end{array}$$

where *l*/*v*_*g*_ corresponds to the propagation time from one side of the cavity to the other. In this new relative time, the pulse propagation along one round trip occurs at the same time, which helps to illustrate the pulse shaping mechanisms in the different sections of the laser.

**Figure 4:**
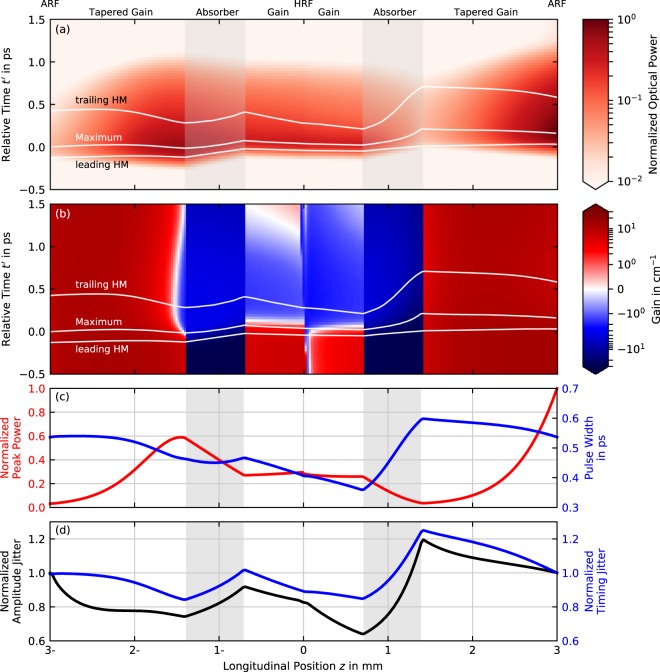
Simulated spatially resolved dynamics in the fundamental mode-locking (FML) regime (*I*_pump_ = 0.81A). The cavity has been unfolded such that the left side of all subplots shows the backwards moving field (denoted with the position suffix -) and the right side shows the forwards moving field. To top row indicates the respective device section and ARF and HRF denote the anti and high-reflection facet. (**a**) shows the pulse shape color-coded over one complete round-trip in the co-moving frame while (**b**) shows the corresponding color-coded evolution of the gain dynamics. Pulse maximum, leading and trailing half-maximum (HM) are indicated with white lines in (**a**,**b**). (**c**) Evolution of the peak power (red) and pulse width (blue), (**d**) the evolution of the amplitude (black) and timing jitter (blue) across the device. (**c**,**d**) Have been obtained from averaging over 20000 round-trips.

In Fig. 4(a), we present the full spatio-temporal pulse evolution along one round-trip in an unfolded cavity, i.e. we show the propagation of *E*^−^(*z*, *t*) from the right out-coupling facet to the high-reflectivity coated facet on the left side of the plot and the propagation of *E*^+^(*z*, *t*) back to the out-coupling facet on the right side of the plot. The pulse power is color coded and normalized to the output power, while the horizontal axis indicates the position within the cavity (note the axis from 3-mm to 0 and back to 3 mm, where the - indicates the backwards traveling pulse) and the vertical axis the relative time *t*′(*z*). As a guide for the eye, the pulse maximum, leading and trailing half-maximum are indicated by white lines. The FWHM corresponds to the vertical distance between the top and bottom line. The corresponding effective optical gain *g*_GS_(2*ρ*^GS^ − 1)/2 − *α*_int_ is plotted in Fig. 4(b), where red colors indicate amplification and blue colors absorption. Additionally, the FWHM of the pulses (blue) and the peak power (red) are averaged over 20000 round-trips and are shown Fig. 4(c).

Starting at the left side of the plot, i.e. the out-coupling facet of the laser, we first follow the backwards moving pulse through the tapered gain section, where the pulse is amplified. As the width of the tapered gain medium decreases in this direction, the number of QDs per given section reduces and the gain saturates easier. This produces an asymmetry in the amplification of the leading and trailing edge of the pulse, which can be seen between 2-mm and 1.4-mm in Fig. 4(b). Especially just before the pulse enters the absorber section, it carries enough energy such that the pulse front alone fully bleaches the gain and the trailing edge is reduced in power by waveguide losses. This firstly leads to a reduction of the pulse FWHM (white lines in Fig. 4(a,b) and blue line in Fig. 4(c)) and secondly to a slight shift of the pulse maximum to earlier times. Upon entering the absorber section, this mechanism reverses: although the pulse does not carry enough energy to completely bleach the absorber, the absorption at and especially past the pulse maximum is significantly weaker, which pushes the pulses maximum to later times and causes a slight rebroadening of the pulse FWHM. The pulse, which then enters the short gain section at the left side of the device, still carries enough energy to easily bleach the quantum dots. The same mechanism as in the narrow part of the tapered gain section leads to a reduction of the pulse width and again shifts the pulse maximum to later times. The pulse peak power remains almost constant in the short gain section, since gain and waveguide losses nearly balance each other.

Upon being reflected at the back facet of the laser as seen in the middle of Fig. 4(a), the pulse travels back through the straight gain section, where the ultra fast carrier relaxation from the QD excited state to the ground state^39,40^, has restored the gain everywhere except right next to the facet. There, waveguide losses still dominate, as indicated by the small vertical blue region in the middle of Fig. 4(b). Along its return through the device, the forwards moving pulse is further shortened by the interplay of saturable gain and waveguide losses, while the peak power stays roughly constant (Fig. 4(c) from *z* = 0.0 to *z* = 0.7). Entering the absorber section, the forwards moving pulse carries less energy compared to the backwards moving pulse and therefore the pulse front as well as the pulse maximum are reduced before the absorber saturates. This results in a strong increase of the pulse FWHM from ≈370 fs to ≈620 fs, which is accompanied by a significant shift of the pulse maximum to later times. Finally, the right moving pulse enters the tapered gain section again, where the increase of QDs along the taper prevents the saturation of the gain and therefore ensures an optimal amplification of the pulse (increasing peak power) before reaching the out-coupling facet. Furthermore, the pulse FWHM reduces from ≈620 fs to ≈560 fs along the tapered gain section.

Our averaging procedure also gives us access to the evolution of the amplitude (black) and timing jitter (blue), which are normalized to their out-coupled values and plotted in Fig. 4(d). We observe that amplitude and timing jitter improve in the gain sections and deteriorate in the absorber sections. This behavior is related to the shift of the pulse maximum (see Fig. 4(a,b)), via the recovery process of the gain and absorption. If a pulse comes slightly too early with respect to the previous pulse in a gain section, the available gain is slightly smaller and the pulse undergoes a smaller shift to earlier times. If the pulse comes slightly too late, the shift of the pulse is stronger and therefore the gain sections naturally counteract perturbations of the pulse position by always pulling the pulses towards their equilibrium position. However in the absorber section, the pulse shifting mechanism works exactly in the other direction and thereby amplifies perturbations of the pulse position. A similar argument applies to the evolution of the amplitude jitter: A pulse carrying slightly more energy than the previous pulse will experience less amplification (reducing the perturbation) and less absorption (amplifying the perturbation) during the next round-trip and vice versa.

In conclusion, we find that pulses broaden in the absorber section and shorten in the gain sections in our device, which is contrary to the common understanding of the pulse-shaping mechanism in semiconductor mode-locked lasers^1,34,50,51^. Specific to our device, the short gain section to the left does not contribute to the out-coupled power, but rather functions as a pulse-shortening section. Moreover, due to the intrinsic self-stabilization mechanism against perturbations in the pulse position and power, the short gain section also improves the pulse train stability and thereby largely contributes to the outstanding performance of this device. To affirm this conclusion, we numerically simulate a device where we exchange the short gain section with an entirely passive section, i.e. turn off the light-matter interaction in that section, but preserve the features of the resonator and increase the gain coefficient of the tapered gain section to maintain the lasing threshold. As a result, we obtain significantly broader pulses (≈900 fs vs. ≈530 fs) and much more pronounced timing jitter (≈30 fs vs. ≈14 fs) and amplitude jitter (≈7.0% vs. ≈4.5%). This confirms the stabilizing properties of the short gain section in the investigated device.

### Influence of the Taper Angle

**Figure 5:**
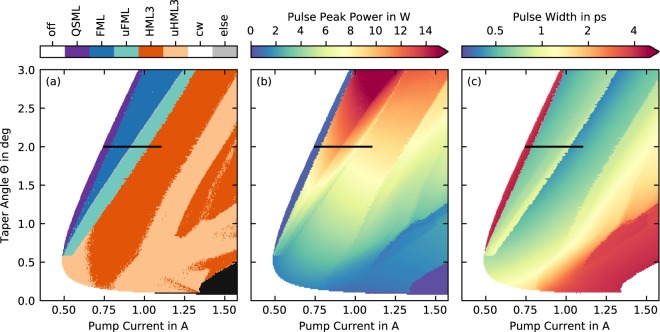
Simulated influence of the taper angle. (**a**) Dynamics observed during laser operation while increasing the pump current for taper angles between Θ = 0° and Θ = 3°. Fundamental mode-locking (FML), unstable fundamental mode-locking (uFML), third-order harmonic mode-locking (HML3), unstable third-order harmonic mode-locking (uHML3), Q-switched mode-locking (QSML) and continuous-wave (cw) are observed and color coded. (**b**) peak output power and (**c**) pulse width obtained from intensity auto-correlations. The black lines in all plots indicate the parameter range shown in Fig. 2.

To investigate the influence of the taper angle, we simulate scans of the pump current for taper angles between Θ = 0° and Θ = 3°. The results are presented in Fig. 5, where color-coded maps of the emission dynamics, the peak power and the pulse width are shown.

Focusing on the dynamics plotted in Fig. 5(a), we find that the sequence of operating states, the laser goes through as we increase the pump current, fundamentally changes with the taper angle. At taper angles close to Θ = 0.0°, no lasing at all is observed as the overall waveguide losses are to large. In a very small region around Θ = 0.1°, cw-lasing (black region in Fig. 5(a)) is found with a high threshold at around *P* ≈ 1.33A. For an increasing taper angle between 0.1° and 0.58°, the threshold quickly reduces to a minimum of *P* ≈ 0.48A, as the waveguide losses of the long tapered section decrease. In this region, the laser first emits unstable third order harmonic mode-locking (uHML3, light orange region in Fig. 5(a)) immediately above threshold. At pump currents of around 0.65 A, these pulse trains transition to a window of stable third order harmonic mode-locking (HML3, orange Figs 5(a), cf. 3(d)), which disappears between *P* ≈ 0.8A and *P* ≈ 1.0A as the laser returns to uHML3 emission with small pockets of HML3 emission. At pump currents around *P* ≈ 1.5A, the laser transitions to cw-emission. In general, the non-cw emission in this small taper-angle region can be characterized by having three pulses circulate in the cavity, which is to be expected as the absorber is roughly placed at one-third of the cavity, leading to colliding pulse mode-locking.

For taper angles above Θ = 0.58°, the behavior of the laser drastically changes. Firstly, the lasing threshold shifts to higher pump currents (*P* ≈ 1A for Θ = 3.0°) for increasing taper-angles, since the increased active region leads to a reduced pump-current density, which results in a decreased saturation of the quantum dots. Secondly, after crossing the threshold, instead of uHML3 or HML3, Q-switched mode-locking (QSML, purple region in Fig. 3(a)) is observed and is followed by fundamental mode-locking (FML, blue region in Fig. 3(b)). The pump-current range of stable fundamental mode-locking increases with the taper angle from a few mA at *θ* = 0.58° to about 230 mA at Θ = 3.00°. Further increasing the pump currents results in a leading edge instability, producing unstable fundamental mode-locking (uFML, cyan, cf. 3(c)). This region is then followed by HML3, which connects to the region of HML3 that is produced at Θ < 0.58°. We explain the occurrence of fundamental-mode locking in a device where a colliding pulse-mechanism should favor third order harmonic mode-locking by the asymmetry in the gain-saturation energies of the left straight gain section and the right tapered section. As the saturation energy is proportional to the number of quantum dots^31^ of a section, increasing the taper angle directly increases the saturation energy asymmetry in the device. We therefore conclude that for the given parameters, a taper angle of at least Θ = 0.58° is required to be able to observe fundamental mode locking.

Furthermore, the peak power and the pulse width are plotted in Fig. 5(b,c), respectively. Pulses are generally the shortest at the lower pump current stability boundary of the stable mode-locking regions. With no substantial dependence on the taper angle, we observe ≈500 fs pulses at the onset of FML emission and ≈470 fs pulses at the onset of HML3 emission. Similarly, but not plotted, the timing and amplitude jitter values for the FML and HML3 region do not change with the taper angle and remain close to the values reported in Sec. 3.1 where a full taper angle Θ = 2.0° was chosen. The highest achievable peak power, however, does critically depend on the taper angle, where we observe the highest values at the upper pump current stability boundary of the FML region (see Fig. 5(b)). Within the FML region, the maximum peak power increases with the taper angle. While we observe ≈10W (experiment and simulation) with a taper angle of 2°, we predict only ≈5 W at a taper angle of Θ = 0.6° and up to ≈15W peak power at a taper angle of Θ = 3°.

### Influence of the Saturable Absorber Section Position

**Figure 6:**
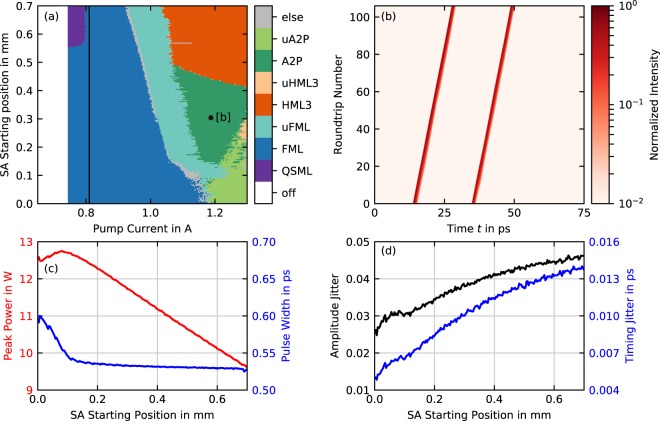
Simulated influence of the saturable absorber position. (**a**) Dynamic regimes of the tapered laser where Q-switched mode-locking (QSML), fundamental mode-locking (FML), unstable FML (uFML), third-order harmonic mode-locking (HML3), unstable HML3 (uHML3), asymmetric two-pulse mode-locking (A2P) and unstable A2P (uA2P) are observed. (**b**) Space-time diagram of A2P at the parameter point indicated with [b] in (**a**). The black vertical line in (**a**) indicates a 1D cut, for which peak power (red) and pulse width (blue) are shown in (**c**) and amplitude (black) and timing jitter (blue) are shown in (**d**).

Lastly, we study the influence of the saturable absorber (SA) position within the three-section cavity. Using our numerical model, we perform scans of the pump current for SA starting positions from ${z}_{{\rm{SA}}}^{{\rm{s}}}=0.0$ mm to ${z}_{{\rm{SA}}}^{{\rm{s}}}=0.7$ mm, while keeping the length of the absorber section constant at 0.7 mm. The former configuration with ${z}_{{\rm{SA}}}^{{\rm{s}}}=0.0$ mm corresponds to the traditional two-section design with the saturable absorber at the highly reflecting end of the cavity, while the latter configuration with ${z}_{{\rm{SA}}}^{{\rm{s}}}=0.7$ mm corresponds to the configuration we study in Sec. 3.1–3.3. The resulting emission dynamics for different absorber positions as a function of the pump current are shown in Fig. 6(a), where the observed mode-locking states are again depicted color-coded as in Fig. 5(a). While the previously described dynamics are seen in the top part of Fig. 6(a), we find two new states for SA starting positions below ${z}_{{\rm{SA}}}^{{\rm{s}}}=0.5$ mm, which are colored in green. Both regimes exhibit two pulses circulating in the cavity, but contrary to second order harmonic mode-locking the pulses have different inter-pulse spacings. We therefore refer to them as asymmetric two-pulse states (A2P, dark green regions in Fig. 6). Similar pulse emission was also found in a two-section quantum well based mode locked laser^52^ and in V-shaped external cavities^53^. They are found at pump currents above *P* ≈ 1.1A for SA starting positions between ${z}_{{\rm{SA}}}^{{\rm{s}}}=0.15$ mm and ${z}_{{\rm{SA}}}^{{\rm{s}}}=0.5$ mm. Below ${z}_{{\rm{SA}}}^{{\rm{s}}}=0.15$ mm unstable asymmetric two-pulse states (uA2P, light green regions in Fig. 6) are observed, which are similar to the uFML emission discussed in Fig. 3(c), but with two adjacent pulses in the cavity that switch their position together. While the lasing threshold remains constant under spatial shifts of the absorber section, Q-switched mode-locking as the first mode-locking state occurs only for SA starting positions from ≈0.55 mm to 0.7 mm. Below ${z}_{{\rm{SA}}}^{{\rm{s}}}\approx 0.55$ mm the laser emits fundamental mode-locking right after the threshold and thereby increases the pump current range for stable FML. The upper stability boundary of the FML regime increases for a decreasing SA starting position from ≈0.91 A at ${z}_{{\rm{SA}}}^{{\rm{s}}}=0.7$ mm to almost 1.2 A at ${z}_{{\rm{SA}}}^{{\rm{s}}}=0.0$ mm, which results in a increase of the FML range from 110 mA at ${z}_{{\rm{SA}}}^{{\rm{s}}}=0.7$ mm to almost 450 mA at ${z}_{{\rm{SA}}}^{{\rm{s}}}=0.0$ mm. Figure 6(b) exemplifies the asymmetric two-pulse state with a space-time diagram for the parameter set indicated with a black dot in Fig. 6(a). The pulse spacings in that case are ≈21.2 ps and ≈54.0 ps, meaning the pulses meet inside the absorber section, thus indicating a colliding pulse effect. As the absorber section is shifted towards ${z}_{{\rm{SA}}}^{{\rm{s}}}=0.0$ mm, the pulse spacings change to ≈14 ps and ≈61.2 ps.

To examine the influence of the SA position on the pulse performance, we plot the peak power (red) and pulse width (blue) in Fig. 6(c) and the amplitude (black) and timing jitter (blue) in Fig. 6(d) along the black line of constant pump current in Fig. 6(a). Decreasing the SA starting position from ${z}_{{\rm{SA}}}^{{\rm{s}}}=0.7$ mm to ${z}_{{\rm{SA}}}^{{\rm{s}}}\approx 0.1$ mm, the peak power increases by ≈3.1W from ≈9.6W to ≈12.7, while the pulse width remains almost constant. Further decreasing the SA starting position to ${z}_{{\rm{SA}}}^{{\rm{s}}}=0.0$ mm however, leads to a increase of the pulse width from ≈530 fs to ≈600 fs, which causes a rollover of the peak power. This behavior is caused by the involved time scales of the laser: For a SA starting position closer to the cavity back facet, the absorber has less time to recover before a reflected pulses reenters and thus less photons are absorbed leading to a greater peak power. Up to ${z}_{{\rm{SA}}}^{{\rm{s}}}\approx 0.1$ mm, the pulse shorting in the straight gain sections occurs to the left and to the right of the absorber section. For ${z}_{{\rm{SA}}}^{{\rm{s}}}\, < \,0.1$ mm however, the right moving pulse that leaves the absorber is to weak to fully saturate the gain section and therefore the pulse shortening mechanism is reduced in its impact, leading to a broadening of the pulses at the out-coupling facet. Amplitude and timing jitter both improve when shifting the saturable absorber towards the back-facet. The amplitude jitter reduces from ≈4.5% to ≈2.6% and the timing jitter from ≈14 fs to ≈5 fs, with a small kink in the curve at ${z}_{{\rm{SA}}}^{{\rm{s}}}\,\approx \,0.1$ mm due to the rollover of the peak power. We attribute this improvement again to the reduced absorber recovery time, which reduces the deterioration of the jitter for the right moving pulse within the absorber section.

Based on these simulations, we predict an optimum performance for a saturable absorber starting position at ${z}_{{\rm{SA}}}^{{\rm{s}}}\,\approx \,0.1$ mm. This configuration might reduce the amplitude and timing jitter by about 50%, increases the peak power by about 30% and avoids pulse-width broadening, that is seen for smaller SA starting positions.

## Conclusion

We studied experimentally and by simulations the pulsed emission dynamics of a semiconductor quantum dot based three section tapered passively mode-locked laser. We demonstrated stable optical pulse train generation across a 90 *mA* wide pump current range with highest pulse peak powers of 10 W with 500 fs short optical pulses and a pulse-to-pulse timing jitter below 100 fs. By combining a traveling-wave equation for the field propagation with the Maxwell-Bloch equations for the field-matter coupling and microscopically motivated rate equations for the electronic degrees of freedom, we derived a model, which excellently reproduces the experimental results. Using the numerical simulations, we have performed an in-depth analysis of the spatio-temporal dynamics in the FML regime. We identified an uncommon pulse-shaping mechanism contrary to the established understanding, where due to the interplay high pulse powers, saturable gain and absorption and waveguide losses, the pulses broaden in the absorber section and shorten in gain section. Hence, the placement of a short gain section between the high-reflective facet and the absorber functions as a pulse-shortening section and is therefore of great importance for the observed outstanding pulse performance. Performing further simulations, we showed that for the absorber position of the experimentally investigated device, fundamental mode-locking is only observed for a sufficient gain saturation energy asymmetry between the two gain sections, which is achieved by the tapered gain structure in our device. If the saturation energy asymmetry is to small, no fundamental mode-locking is observed as the laser favors third order harmonic mode-locking via a colliding-pulse mechanism. Shifting the saturable absorber closer to the high-reflective facet also breaks the third order colliding pulse mode-locking and increases the range of stable fundamental mode-locking. Furthermore, an optimal saturable absorber position was found, which maximizes the output power and minimizes the amplitude and timing-jitter while retaining the ultra short pulses. Based on these results, we predict that tuning the taper angle and saturable absorber position has the capability of improving the already outstanding performance of the presented device. Optimizing the other device parameters has the potential of pushing the achievable performance even further. Chasing this goal, our proposed numerical model is efficient enough to implement large parameter studies and thereby guide the design of monolithically integrated mode-locked semiconductor lasers.

## Methods

### Model and Numerical Simulations

In order to gain an in-depth understanding of the observed characteristics and study the implications of the device design, we aim to first reproduce the measurements by numerical simulations. Hence, we require a model that is capable of describing the device specific spatio-temporal evolution of the electric field within the quantum dot active medium. However, we also need our simulations to be numerically efficient enough to calculate sufficiently long time series for the evaluation of timing and amplitude jitter as well as to perform multi-parameter studies. The well established delay-differential equation modeling approaches^54–56^ are computationally very efficient, but require limiting assumptions about the device geometry and spectral field evolution. We therefore follow the ideas of ^35,49,57,58^ and propose a model that couples a traveling-wave equation for the propagation of the electric field via effective Maxwell-Bloch equations to microscopically motivated rate equations that describe the electronic degrees of freedom in the active medium. To achieve the required computational efficiency, we describe the quantum dots by averaging over the inhomogeneously broadened quantum dots^35,40,59^ and neglect the effects of spatial hole burning^57,58^. In the following, we present the derivation of our model.

**Figure 7:**
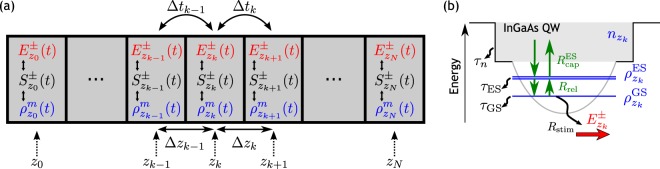
(**a**) Pulse propagation scheme. The left (−) and right (+) traveling waves *E*^±^ in one section *k* are coupled to the respective carrier population *ρ*^*m*^ via the field source term *S*^±^ and to neighboring sections via the propagation time Δ*t*_*k*_. (**b**) Sketched quantum dot conduction band structure with carrier interaction processes. Only the quantum dot ground state couples to the in-phase gain of the electric field.

The field propagation is described in the slowly-varying envelope and rotating wave approximation, which yield first order partial differential equations for the right (+) and left (−) moving traveling-wave envelope function *E*^±^(*z*, *t*)^60^

2 $$\begin{array}{ccc}(\pm {\partial }_{z}+\frac{1}{{v}_{g}}{\partial }_{t})\,{E}^{\pm }(z,t) & = & \frac{i\omega {\rm{\Gamma }}}{2{\varepsilon }_{b}{v}_{g}}{P}^{\pm }(z,t)={S}^{\pm }(z,t)\end{array}$$

where *P*^±^ is the active medium macroscopic polarization, *ω* the optical center frequency, Γ the geometrical confinement factor, *v*_*g*_ the group velocity and *ε*_*b*_ the background permittivity, which are summarized by the source term *S*^±^. Integrating this equation along its characteristic curve and using the trapezoid approximation for the RHS with the inclusion of waveguide losses *α*_int_ yields the delay algebraic field propagation scheme

3 $$\begin{array}{ccc}{E}_{k}^{\pm }(t) & = & \frac{4-{\rm{\Delta }}z{\alpha }_{\mathrm{int}}}{4+{\rm{\Delta }}z{\alpha }_{\mathrm{int}}}\,{E}_{k\mp 1}^{\pm }(t-{\rm{\Delta }}t)+\frac{{\rm{\Delta }}z}{2+{\rm{\Delta }}z{\alpha }_{\mathrm{int}}}[{S}_{k\mp 1}^{\pm }(t-{\rm{\Delta }}t)+{S}_{k}^{\pm }(t)]\end{array}$$

for an equidistant discretization Δ*z* and Δ*t* = Δ*z*/*v*_*g*_, where Δ*t* corresponds to the propagation time between two adjacent sections. This approach allows for a much coarser spatial discretization while maintaining a high temporal accuracy^61,62^. The pulse propagation scheme is sketched in Fig. 7(a) for a discretization of *N* sections at the positions *z*_*k*_, *k* ∈ {1, ..., *N*}. The boundary conditions are given by the reflection of the electric field at the left and right facets of the cavity with the intensity reflectivity coefficients *κ*_L_ and *κ*_R_.

The active medium polarization is calculated semi-classically and is determined by the sum of all microscopic polarization amplitudes ${p}_{\alpha }^{\pm }$ where *α* denotes a suitable set of quantum numbers. We assume that only the ground and first excited state quantum dot transitions contribute to the optical gain and their transition frequencies are not subject to inhomogeneous broadening. As a result, the sum reduces to a simple multiplication with the quantum dot sheet density *N*^QD^

4 $${P}^{\pm }=\frac{2}{{V}_{{\rm{act}}}}\,{\sum }_{\alpha }{\mu }_{\alpha }^{\ast }{p}_{\alpha }^{\pm }=4\frac{{N}^{{\rm{QD}}}}{{h}^{{\rm{QW}}}}({\nu }_{{\rm{GS}}}{\mu }_{{\rm{GS}}}^{\ast }{p}_{{\rm{GS}}}^{\pm }+{\nu }_{{\rm{ES}}}{\mu }_{{\rm{ES}}}^{\ast }{p}_{{\rm{ES}}}^{\pm })$$

where *V*_act_ denotes the active medium volume, *h*^QW^ the height of the surrounding quantum well reservoir, *μ*_GS,ES_ the respective dipole moment and *ν*_GS,ES_ the relative degeneracy of the GS and ES. The dynamics of the microscopic polarization amplitudes of the ground and excited state (*m* ∈ {GS, ES}) are given by the Maxwell-Bloch equations

5 $$\frac{d}{dt}{p}_{{\rm{m}}}^{\pm }=-\,[i{\rm{\Delta }}{\omega }_{{\rm{m}}}+\frac{1}{{T}_{2}}]\,{p}_{{\rm{m}}}^{\pm }-\,i\frac{{\mu }_{{\rm{m}}}}{2\hslash }{E}^{\pm }(2{\rho }^{{\rm{m}}}-1)$$

where Δ*ω*_m_ is the detuning from the optical center frequency and *T*_2_ the effective polarization dephasing time, which is assumed to be equal for the ground and excited state, and the excitonic quantum dot occupation probability *ρ*^*m*^. Note that the effective dephasing time *T*_2_ reflects the full gain bandwidth of the GS(ES) ensemble and not the homogeneous linewidth of an individual optical transition, as we have averaged over the inhomogeneous broadening. Under the assumption that *ρ*^m^ evolves slowly compared to ${p}_{{\rm{m}}}^{\pm }$, Eq. (5) can be formally solved and written as^35,57^

6 $${p}_{{\rm{m}}}^{\pm }=-i\frac{{\mu }_{{\rm{m}}}{T}_{2}}{2\hslash }(2{\rho }^{{\rm{m}}}-1)\,{G}_{{\rm{m}}}^{\pm }$$

with the new variable ${G}_{{\rm{m}}}^{\pm }$, which behaves as a filtered electric field, whose dynamics are given by

7 $$\frac{d}{dt}{G}_{{\rm{m}}}^{\pm }=\frac{1}{{T}_{2}}({E}^{\pm }-{G}_{{\rm{m}}}^{\pm })+i{\rm{\Delta }}{\omega }_{{\rm{m}}}{G}_{{\rm{m}}}^{\pm }+\sqrt{D{\rho }^{{\rm{m}}}}\xi (t)$$

where the effect of stochastic spontaneous emission has been added via the last term with the *δ*-correlated complex Gaussian white noise *ξ*(*t*) and the noise strength *D*, which is tuned to match the experiment. Assuming the electric field frequency is centered at the GS transitions, we adiabatically eliminate the dynamical equation for ${G}_{{\rm{ES}}}^{\pm }$ and obtain ${G}_{{\rm{ES}}}^{\pm }={\mathrm{(1}+i{T}_{2}{\rm{\Delta }}{\omega }_{{\rm{ES}}})}^{-1}\,{E}^{\pm }$, which has a vanishing real part compared to the imaginary part for the given parameters and therefore only contributes to the amplitude-phase coupling. With the help of the differential gain of both transitions, *g*_m_ = 2*ω*Γ*T*_2_*N*^QD^*ν*_m_|*μ*_m_|^2^/*ε*_*b*_ℏ*v*_*g*_*h*^QW^, the electric field source term can be expressed as

8 $${S}^{\pm }=\frac{{g}_{{\rm{GS}}}}{2}\mathrm{(2}{\rho }^{{\rm{GS}}}-\mathrm{1)}{G}_{{\rm{GS}}}^{\pm }-i\frac{\delta {\omega }^{{\rm{E}}S}}{2}(2{\rho }^{{\rm{ES}}}-1){E}^{\pm }$$

where *δω*^ES^ = *g*_ES_*T*_2_Δ*ω*_ES_[1 + (*T*_2_Δ*ω*_ES_)^2^]^−1^ denotes the amplitude-phase coupling due to the ES population. Note that the effective dephasing time *T*_2_ not only determines the gain bandwidth, but also the gain coefficient *g*_m_. Since the optical gain derived from the full semiconductor Bloch-equations depends upon the microscopic dephasing time of the individual transitions^39,59^, which is not explicitly included in our model, we treat the gain coefficients *g*_m_ that are used for the simulations as fit parameters, which are adjusted to best match the experiment.

The charge-carrier model includes the excitonic occupation numbers of the quantum dot ground and excited state and the charge-carrier density in the surrounding quantum well. Figure 7(b) shows a sketch of the different levels and their interaction via scattering processes. Their dynamics at each spatial coordinate are described by a set of coupled rate equations^40^

9 $$\frac{d}{dt}n=-\,\frac{n}{{\tau }^{n}}+J-4{N}^{{\rm{QD}}}{R}_{{\rm{cap}}}^{{\rm{ES}}}$$

10 $$\frac{d}{dt}{\rho }^{{\rm{ES}}}=-\,\frac{{\rho }^{{\rm{ES}}}}{{\tau }^{{\rm{ES}}}}+{R}_{{\rm{cap}}}^{{\rm{ES}}}-\frac{1}{2}{R}_{{\rm{rel}}}$$

11 $$\frac{d}{dt}{\rho }^{{\rm{GS}}}=-\,\frac{{\rho }^{{\rm{GS}}}}{{\tau }^{{\rm{GS}}}}+{R}_{{\rm{rel}}}-{\partial }_{t}{\rho }^{{\rm{GS}}}{|}_{{\rm{stim}}}$$

with the pump current density *J*, the characteristic carrier lifetimes *τ*^GS,ES,n^, the factor 4 for spin and ES degeneracy, the net carrier capture from the wetting layer ${R}_{{\rm{cap}}}^{{\rm{ES}}}$ and the net intra-dot carrier relaxation *R*_rel_^38,63,64^. The net intra-dot relaxation

12 $${R}_{{\rm{rel}}}={\tilde{R}}_{{\rm{rel}}}[\mathrm{(1}-{\rho }^{{\rm{GS}}}){\rho }^{{\rm{ES}}}-{\rho }^{{\rm{GS}}}\mathrm{(1}-{\rho }^{{\rm{ES}}}){e}^{-\frac{{\rm{\Delta }}{\varepsilon }^{{\rm{ESGS}}}}{{k}_{B}T}}]$$

includes Pauli-blocking terms and a Boltzmann-factor with the energy difference Δ*ε*^ESGS^ between the QD excited and ground state and the effective temperature *T* to to account for detailed balance between the in and out-scattering processes. Thereby, relaxation towards the quasi-equilibrium is ensured. The net carrier-capture rate is given by

13 $${R}_{{\rm{cap}}}={\tilde{R}}_{{\rm{cap}}}[\frac{1}{1+{e}^{{\rm{\Delta }}{\varepsilon }^{{\rm{QWES}}}/{k}_{B}T}/({e}^{n/{D}^{{\rm{2}}D}{k}_{B}T}-\mathrm{1)}}-{\rho }^{{\rm{ES}}}]$$

where the left term inside the parenthesis describes the carrier density dependent quasi-Fermi function with the quantum-well band edge to QD excited state energy difference Δ*ε*^QWES^ and the two-dimensional density of states *D*^2D^ ^65^. The coupling to the electric field is embedded in the stimulated emission term in Eq. (11), which takes the form

14 $${\partial }_{t}{\rho }^{{\rm{GS}}}{|}_{{\rm{stim}}}={g}^{{\rm{GS}}}\eta \mathrm{(2}{\rho }^{{\rm{GS}}}-\mathrm{1)}{\rm{Re}}({G}_{{\rm{GS}}}^{+}{E}^{{+}^{\ast }}+{G}_{{\rm{GS}}}^{-}{E}^{{-}^{\ast }}).$$

with the photon to field conversion factor *η* = *ε*_*b*_*v*_*g*_*h*^QW^/(4ℏΓ*N*^QD^).

So far, we have modeled the charge-carrier dynamics withing the gain sections where a forward bias is applied. In the absorber section however, a reverse bias *U* is applied, whose effect is twofold. Firstly, the static transverse electric field reduces the barrier height, which leads to enhanced thermionic carrier escape rates^66,67^. Following^68^, this is implemented by an effective ES lifetime ${\tau }_{{\rm{abs}}}^{{\rm{ES}}}(U)$, which exponentially depends on the absorber bias *U*. Secondly, the optical transitions are slightly red shifted due to the quantum confined stark effect^66,69^, which is implemented via the detuning Δ*ω*_GS_ in the polarization equation. We assume that the red shift scales linearly with the applied reverse bias. The constituting equations are then given by

15 $${\tau }_{{\rm{abs}}}^{{\rm{ES}}}(U)={\tau }_{{\rm{abs}}\mathrm{,0}}^{{\rm{ES}}}\,\exp \,(U/{U}_{0}^{{\tau }_{{\rm{abs}}}^{{\rm{ES}}}})$$

16 $${\rm{\Delta }}{\omega }_{{\rm{GS}}}^{{\rm{abs}}}(U)={\rm{\Delta }}{\omega }_{{\rm{GS}}\mathrm{,0}}^{{\rm{abs}}}U$$

where the parameters are chosen similarly to^66,68,69^ and are given in Table 1. Additionally, we set the differential gain coefficient in the absorber section to ${g}_{{\rm{GS}}}^{{\rm{abs}}}\, > \,{g}_{{\rm{GS}}}^{{\rm{gain}}}$, since the decreased carrier density in the surrounding quantum well leads to reduced coulomb scattering and thereby to an increased microscopic dephasing time of the optical transitions^70^.

**Table 1:** Parameters used in the numerical model.

Symbol	Value	Symbol	Value	Symbol	Value
*α*_int_	6 cm^−1^	*N* ^*QD*^	0.2*10^11^ cm^−2^	*ν*_GS_, *ν*_ES_	1,2
$2\hslash /{T}_{2}^{{\rm{gain}}}$	50 meV	${g}_{{\rm{GS}}}^{{\rm{gain}}}$	30 cm^−1^	$\delta {\omega }_{{\rm{gain}}}^{{\rm{ES}}}$	17.4 cm^−1^
$2\hslash /{T}_{2}^{{\rm{abs}}}$	50 meV	${g}_{{\rm{GS}}}^{{\rm{abs}}}$	60 cm^−1^	$\delta {\omega }_{{\rm{abs}}}^{{\rm{ES}}}$	34.8 cm^−1^
*κ*_L_	0.95	${\tilde{R}}_{{\rm{rel}}}$	5 ps^−1^	${\tilde{R}}_{{\rm{cap}}}$	0.1 ps^−1^
*κ*_R_	0.03	Δ*ε*^ESGS^	64 meV	Δ*ε*^QWES^	24 meV
${\rm{\Delta }}{\omega }_{{\rm{GS}}\mathrm{,0}}^{{\rm{abs}}}$	0.367 THzV^−1^	${\tau }_{{\rm{abs}}\mathrm{,0}}^{{\rm{ES}}}$	20 ps	${U}_{0}^{{\tau }^{{\rm{ES}}}}$	2.0 V
*τ* ^*n*^	1 ns	*τ* ^ES^	1 ns	*τ* ^GS^	1 ns
${\alpha }_{{\rm{int}}}^{{\rm{T}}}$	4.8 cm^−1^	Γ_T_	0.25	*a*_L_	10
*w*_0_	4 *μ*m	*D*^2D^ℏ^2^	6.96*10^−32^ kg	*γ*	0.3

Lastly, the effect of the taper gain structure has to be included in the model. On that account, we assume that the transverse electric field follows the profile of the active region by adiabatically expanding and reducing in its spatial extend^31,37,71^. In our model, this is described by rescaling the stimulated emission term in Eq. (11) with the relative change of the ridge width *w*(*z*)

17 $${\partial }_{t}{\rho }^{{\rm{GS}}}(z{)|}_{{\rm{stim}}}^{{\rm{taper}}}=\frac{{w}_{0}}{w(z)}{\partial }_{t}{\rho }^{{\rm{GS}}}(z{)|}_{{\rm{stim}}}$$

where *w*_0_ is the width of active region without taper. As the lateral electric field profile is distributed over a larger area in the tapered region, the quantum dots see only a reduced field strength, depending on the local waveguide width, *w*(*z*). This results in a reduced stimulated recombination rate and thus a higher saturation energy of the active medium.. Additionally, we assume that increasing the ridge width leads to better overlap of the field with the active medium, thus improving the transverse confinement factor Γ(*z*), while simultaneously reducing the the waveguide losses *α*_int_(*z*)^35^. These effects are modeled phenomenologically by the fit functions

18 $${\alpha }_{{\rm{int}}}(z)={\alpha }_{{\rm{int}}}^{0}-{\alpha }_{{\rm{int}}}^{{\rm{T}}}tan\,h\,(\frac{{w}_{0}-w(z)}{{w}_{0}}-1)$$

19 $${{\rm{\Gamma }}}_{{\rm{rel}}}(z)=1+{{\rm{\Gamma }}}_{{\rm{T}}}tan\,h\,(\frac{{w}_{0}-w(z)}{{w}_{0}}-1)$$

where the fit parameter Γ_T_ and the fit function Eq. (19) is chosen to mimic results from beam-propagation calculations^35,71,72^ and ${\alpha }_{{\rm{int}}}^{{\rm{T}}}$ and the fit function Eq. (18) is chosen the match waveguide characterization measurements.

Finally, the pump current *P* is related to the pump current density *J* via

20 $$P=J{A}_{{\rm{G}}}{a}_{{\rm{L}}}e{\gamma }^{-1}$$

where *A*_G_ is the area of the active region, *a*_L_ the number of QD layers, *e* the electron charge and *γ* the injection effiency, which is fitted to the experimental data. The out-coupled power is calculated according to

21 $${P}_{{\rm{out}}}=2\hslash \omega {a}_{{\rm{L}}}{w}_{0}{N}^{{\rm{QD}}}\eta {|{E}_{{\rm{out}}}|}^{2}$$

where the out-coupled electric field at the right facet of the tapered section is given by |*E*_out_|^2^ = (1 − *κ*_R_)|*E*(*z* = *l*)|^2^. After an initial transient time (typically only between 20 and 50 round-trips due to the low right mirror reflectivity), the relevant figures of merit can then be calculated either directly from time series or from numerically computed auto-correlation functions. The amplitude is quantified by the standard deviation of the pulse-peak powers and normalized to the mean. The timing jitter is measured by the standard deviation of the inter-pulse intervals, which corresponds to the pulse-to-pulse or period jitter. Contrary to the long-term timing-jitter, this characterization includes the short-term correlations, which are introduced by the recovery processes of the gain and absorber sections^20^. Hence, changes of the laser design, which affect the dynamics of the gain and absorber sections are also made visible in the pulse-to-pulse timing-jitter.

In summary, we have derived a system of coupled differential-delay-algebraic equations (DDAEs) Eqs (3, 8–11), which describe the spatio-temporal evolution of the electric field and the charge-carrier populations in the laser. Direct integration of these equations produces time-series of all dynamical variables to be used for analysis and characterization. The integration is implemented via a fourth-order Runge-Kutta approach, that converges for our system with a time-step of 30 fs and a spatial discretization of 50 *μ*m, i.e. 60 sections along the device. On a state of the art desktop CPU (Intel Core i7-4770), a 75 *μ*s time-series (about 1000 round-trips) takes roughly two minutes to compute and already produces reasonable pulse-train statistics. A list of all simulations parameters and their values is presented in Table 1.
